# Human Alveolar Epithelial Cell Injury Induced by Cigarette Smoke

**DOI:** 10.1371/journal.pone.0026059

**Published:** 2011-12-07

**Authors:** Beata Kosmider, Elise M. Messier, Hong Wei Chu, Robert J. Mason

**Affiliations:** Department of Medicine, National Jewish Health, Denver, Colorado, United States of America; Beth Israel Deaconess Medical Center, United States of America

## Abstract

**Background:**

Cigarette smoke (CS) is a highly complex mixture and many of its components are known carcinogens, mutagens, and other toxic substances. CS induces oxidative stress and cell death, and this cell toxicity plays a key role in the pathogenesis of several pulmonary diseases.

**Methodology/Principal Findings:**

We studied the effect of cigarette smoke extract (CSE) in human alveolar epithelial type I-like (ATI-like) cells. These are isolated type II cells that are differentiating toward the type I cell phenotype *in vitro* and have lost many type II cell markers and express type I cell markers. ATI-like cells were more sensitive to CSE than alveolar type II cells, which maintained their differentiated phenotype *in vitro*. We observed disruption of mitochondrial membrane potential, apoptosis and necrosis that were detected by double staining with acridine orange and ethidium bromide or Hoechst 33342 and propidium iodide and TUNEL assay after treatment with CSE. We also detected caspase 3 and caspase 7 activities and lipid peroxidation. CSE induced nuclear translocation of Nrf2 and increased expression of Nrf2, HO-1, Hsp70 and Fra1. Moreover, we found that Nrf2 knockdown sensitized ATI-like cells to CSE and Nrf2 overexpression provided protection against CSE-induced cell death. We also observed that two antioxidant compounds N-acetylcysteine and trolox protected ATI-like cells against injury by CSE.

**Conclusions:**

Our study indicates that Nrf2 activation is a major factor in cellular defense of the human alveolar epithelium against CSE-induced toxicity and oxidative stress. Therefore, antioxidant agents that modulate Nrf2 would be expected to restore antioxidant and detoxifying enzymes and to prevent CS-related lung injury and perhaps lessen the development of emphysema.

## Introduction

Cigarette smoke (CS) is a highly complex mixture and many of its components are known carcinogens, mutagens, and other toxic substances. In the United States, over 400,000 deaths per year are attributed to smoking. CS is a complex chemical aerosol composed of particulates suspended in a gaseous phase [1]. The tar particles in the smoke are of different sizes and the smallest particles have the greatest potential to reach deep into the lung. The pulmonary epithelium is the barrier between inhaled air and the underlying tissue. Type I cells compose 95% of the alveolar surface in the distal gas exchange region of the lung. To maintain this barrier, continuous cell replacement and repair of the epithelium are important. Cell death through apoptosis is essential for eliminating damaged cells during tissue remodeling and inflammation. Disturbance of this physiological process can however, result in necrosis or excessive apoptosis with disruption of the barrier function of the epithelium, leading to lung injury and the development of emphysema [2]. Emphysema develops because of destruction of the alveolar wall and damage to the alveolar epithelium and underlying microvasculature. The pathogenesis of emphysema has traditionally focused on the protease imbalance as exemplified by alpha-1-antitrypsin deficiency and the resultant destruction of the extracellular matrix, especially elastin fibers [3], [4]. However, more recently attention has been given to a complementary hypothesis of cellular injury and resultant apoptosis due to oxidants in CS or those induced by cigarette smoke extract (CSE) [5]. The two most sensitive cells to oxidant injury in the alveolar septum are type I alveolar epithelial cells and microvascular endothelial cells. Recent studies have shown an increase in apoptotic cells in the lungs of patients with chronic obstructive pulmonary disease (COPD) [6]. Most of the apoptotic cells were alveolar epithelial cells and not microvascular endothelial cells. The majority of the apoptotic epithelial cells were thought to be alveolar type II (ATII) cells, but this could have been due in part to a sampling error in that type I cells are very large and flat and pathologic sections might easily miss the nucleus of apoptotic type I cells. Currently we know very little about the susceptibility of human alveolar epithelilal cells to CS or the regulation of the antioxidant defenses.

Phase 2 detoxifying enzymes are classified as “indirect” antioxidant enzymes due to their role in redox balance and thiol homeostasis [7], [8], [9]. Nuclear factor-erythroid 2 related factor 2 (Nrf2) belongs to the Cap'n'collar (CNC)-basic leucine zipper (bZIP) transcription factor family. *Nrf2* is constitutively expressed in almost all cell types and tissues but is most abundant in tissues where routine detoxification reactions occur normally, including lung, intestine, and kidney. Under basal conditions, Nrf2 is sequestered in the cytoplasm by Kelch-like ECH-associated protein 1 (Keap1). Nrf2 is known to be activated by phosphorylation *via* several protein kinase pathways leading to Keap1•Nrf2 dissociation and nuclear translocation. Nrf2 exerts its role in host protection against oxidative injury and carcinogenesis *via* binding to *cis*-acting promoter sequences, called antioxidant response elements (AREs), which leads to induction of numerous ARE-bearing antioxidant/defense and cytoprotective genes. Pulmonary *Nrf2* effector genes bearing AREs include phase 2 antioxidant/detoxifying enzymes as well as stress proteins such as heme oxygenase 1 (HO-1) [7], [8], [9]. The alveolar epithelium is exposed to high levels of free radicals which are present in smoke and can damage cellular macromolecules [9]. *HO-1* plays a central role in the defense against lung oxidative and inflammatory insults, including CS exposure [10] and its overexpression protects against oxidative stress [11]. CS enhances expression of Nrf2 and phase 2-related genes regulated by Nrf2. This was shown *in vivo* in mice that lack the Nrf2 gene, which indicates that the activation of Nrf2 may represent a key cytoprotective response mechanism against cell injury induced by CS [12]. It is important to note in this context that Nrf2 targeting might provide clinical benefit by reducing both oxidative stress and inflammation in emphysema [13].

Therefore in this study we focused on two antioxidants N-acetylcysteine (NAC) and trolox that have been found to reduce oxidative stress and inflammation. NAC is a membrane-permeable thiolic compound that contains a sulfhydryl group donor, serving as a precursor of glutathione (GSH) synthesis and inhibits the formation of reactive oxygen species (ROS) [14], [15]. NAC has been shown to exhibit protective effects against DNA oxidative damage by its antioxidant properties [16], [17]. It was also reported that NAC attenuates pulmonary emphysema and alveolar septal cell apoptosis *in vivo* in rats [18] and reduced air trapping in patients with moderate to severe emphysema [19]. Trolox is a water soluble derivative of vitamin E and is part of a family of essential micronutrients comprising lipid-soluble tocopherols and tocotrienols with potent antioxidant activities [20]. This compound has been reported to be an excellent antioxidant *in vitro* [21]. Furthermore, acute exacerbations of emphysema induce oxidative stress [22], which may be decreased by trolox. Therefore we focused in this study on the alveolar cell injury by CSE, the role of Nrf2 and antioxidant defense system.

Although alveolar type I cells are the likely target of oxidants in CS because of their large surface area and sensitivity to oxidant injury, they are difficult to study. Human alveolar type I cells have not been isolated for *in vitro* studies to our knowledge. We have chosen to use alveolar type I-like (ATI-like) cells which are ATII cells that are transitioning to become type I cells in that they no longer express the surfactant proteins, SP-A, SP-B and SP-C but do express type I cell markers caveolin and receptor for advanced glycation end products (RAGE) [23]. The purpose of this project was to determine the susceptibility of ATI-like cells to CSE, the role of Nrf2 in protecting ATI-like cells to injury, and efficiency of antioxidant compounds NAC and trolox to protect ATI-like cells to injury due to CS. There is no previous study on the role of Nrf2 protecting human ATI-like cells. We hypothesize that Nrf2 will play an important role in preventing injury to alveolar epithelial cells by CSE. We also expect that antioxidant compounds NAC and trolox will provide protection.

## Results

### CSE Induces Both Apoptosis and Necrosis in ATI-like Cells

**Figure 1:**
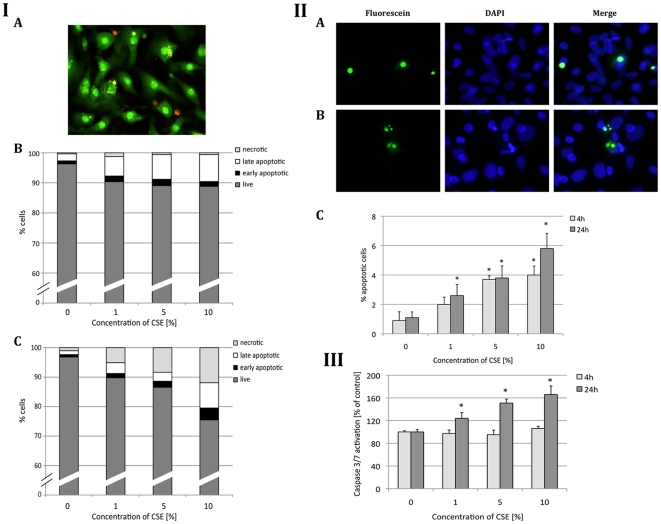
CSE induces apoptosis and necrosis in ATI-like cells. Panel I – Acridine orange and ethidium bromide double staining. A - Cells were analyzed after 4 h treatment with 5% CSE. Live cells are green, late apoptotic cells have chromatin condensation (yellow) and necrotic cells are red; B - Cells were treated with CSE for 4 h; C - Cells were exposed to CSE for 24 h. Data represent results from three independent experiments. Panel II - TUNEL assay. A - Chromatin condensation in cells exposed to 5% CSE for 24 h; B – Chromatin fragmentation after cell treatment with 5% CSE for 24 h; C - ATI-like cells were treated with CSE for 4 h and 24 h. Data represent results from three independent experiments (*p*<0.05). Panel III - Caspase 3 and caspase 7 activities. Cells were treated with CSE for 4 h and 24 h. Data are expressed in percentages of the control (*p*<0.05).

In our initial experiments we compared the toxicity induced by CSE in both differentiated ATII cells and ATI-like cells (Figure S1A, B). As measured by propidium iodine staining, ATI-like cells were significantly more sensitive than ATII cells after treatment with 10% CSE for 24 h. We had found previously the same correlation that ATI-like cells were more sensitive than ATII cells to ozone [24]. We chose the CSE concentrations to study acute toxicity based on ATI-like cell double staining with acridine orange and ethidium bromide to distinguish between live, early and late apoptotic cells and necrotic cells (Figure 1, Panel I, A, B, C). Our time points were chosen on the basis of preliminary data. We found more apoptotic cells than necrotic cells after ATI-like cell treatment with CSE for 4 h and more necrotic cells after 24 h treatment. These results indicate that CSE induces both apoptosis and necrosis in a time- and concentration-dependent manner in ATI-like cells.

We observed chromatin condensation and fragmentation in ATI-like cells after treatment with CSE using TUNEL assay (Figure 1, Panel II, A, B, C). The percentage of apoptotic cells increased in a concentration and time-dependent manner. We observed the highest induction of apoptosis (5.8%) after 24 h treatment with CSE. We found a statistically significant increase in apoptotic ATI-like cells after 24 h exposure to all applied concentrations of CSE and after 4 h of treatment with 5% and 10% CSE. We then used a caspase activity assay to further define the apoptosis pathway induced by CSE (Figure 1, Panel III). These results suggest a caspase-dependent apoptosis pathway induced by CSE in ATI-like cells.

### CSE Induces Lipid Peroxidation in ATI-like Cells

**Figure 2:**
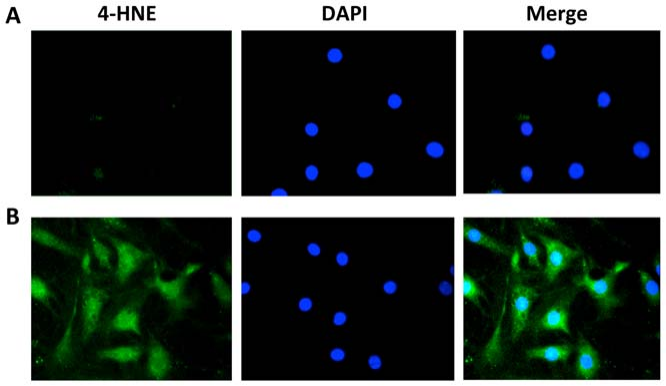
CSE produces 4-HNE in ATI-like cells. A - Control; B - Cells exposed to 5% CSE for 24 h. Cells were probed by 4-HNE primary antibody, stained with Alexa Fluor 488 as secondary antibody (green) and mounted with Vectashield medium containing DAPI (blue). Shown are representative images from three independent experiments.

Immunostaining with 4-HNE, which is a specific and stable product of lipid peroxidation, was used to detect oxidative stress induced by CSE in ATI-like cells (Figure 2A, B). This exposure induced cellular immunocytofluorescence for 4-HNE in comparison with control. We observed an increase in the intensity of cytoplasmic and nuclear staining. These data suggest that CSE increases 4-HNE production as a consequence of oxidative stress, and this observation is consistent with our other results confirming that CSE is a strong activator of ROS-induced protein expression.

### CSE Induces Nrf2 Translocation

**Figure 3:**
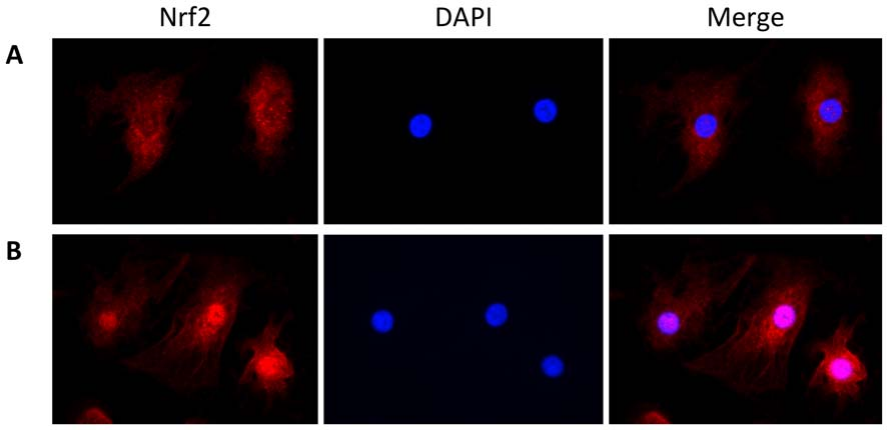
CSE induces nuclear localization of Nrf2 in ATI-like cells as detected by immunocytofluorescence. A - Cytoplasmic localization of Nrf2 in control cells (red stain); B - Nuclear translocation of Nrf2 after 4 h treatment with 5% CSE. Representative data each from one of three experiments are shown.

Because CSE induces cell injury and oxidative stress, we wanted to determine if the main regulator of defense against oxidant stress, the transcription factor Nrf2 was activated. We used immunocytofluorescence to determine the effect of CSE on Nrf2 localization in ATI-like cells. We observed the highest nuclear translocation of Nrf2 between 1.5 h to 4 h after these cells were exposed to 5% CSE in comparison with control (Figure 3A, B). This translocation was lower after cell treatment with 5% CSE for 24 h (data not shown). This observation suggests that CSE induces ATI-like cell antioxidant response through an Nrf2-dependent mechanism but it is not sustained.

### NAC and Trolox Protect ATI-like Cells Against CSE

Because of the sensitivity of ATI-like cells to the cytotoxic effects of CSE (Figure S1), we applied NAC and trolox in ATI-like cells *in vitro.* Also because of their surface area type I cells would be likely the prime targets of CS in the alveolar region. To determine if antioxidant compounds NAC and trolox would block the cell injury induced by CSE, we initially tested the effect on cell viability. We used the MTT assay (Data S1) to determine non-toxic concentrations of NAC and trolox. We found 97.4% and 96.2% viability after 24 h treatment of ATI-like cells with 5 µM NAC or trolox, respectively. Therefore, we selected this concentration for both antioxidant compounds for all subsequent experiments.

**Figure 4:**
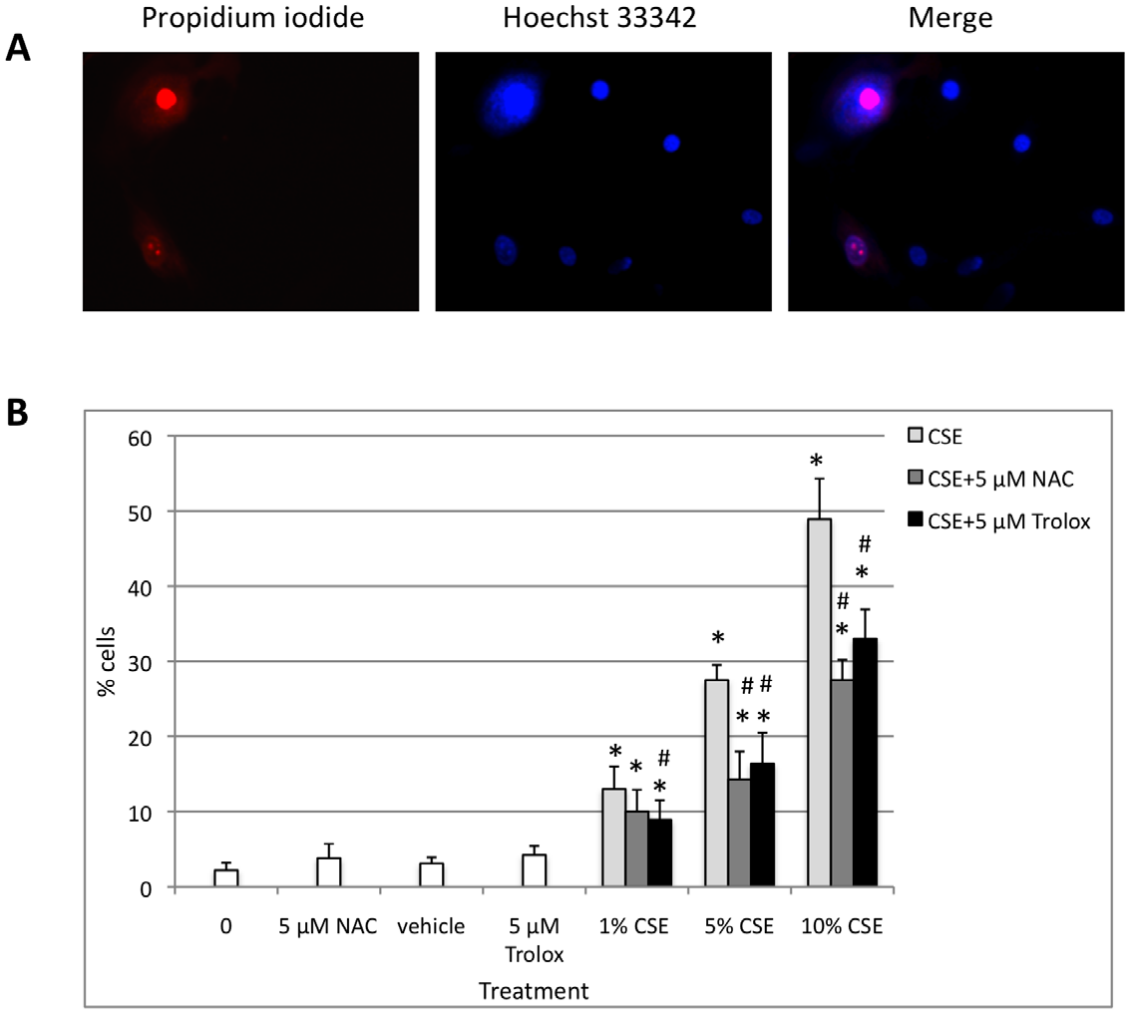
NAC and trolox protect ATI-like cells treated with CSE. Cells were exposed to CSE or co-treated with CSE and NAC or CSE and trolox for 24 h and toxicity was measured by propidium iodide and Hoechst 33342 double staining. A - Necrotic ATI-like cells after treatment with 5% CSE for 24 h. B – Co-treatment with CSE and 5 µM NAC or CSE with 5 µM trolox for 24 h decreases the percentage of necrotic cells in comparison with CSE alone. * - Statistically significant increase in percentage of necrotic cells induced by CSE, co-treatment with CSE and NAC or CSE and trolox in comparison with control, NAC or trolox, respectively. # - Statistically significant decrease of ATI-like necrotic cells after co-treatment with CSE and NAC or CSE and trolox in comparison with cell exposure to CSE. Data represent results from three independent experiments (*p*<0.05).

We found that co-treatment with CSE and 5 µM trolox statistically reduced cell necrosis in comparison with all applied concentrations of CSE. We also found that 5 µM NAC significantly reduced necrosis of ATI-like cells to 5% or 10% CSE (Figure 4A, B). Treatment with either 5 µM NAC or 5 µM trolox prevented the nuclear translocation of Nrf2 due to CSE (data not shown). However, further studies on the exact mechanisms of action of these antioxidant compounds are required.

### CSE Increases Nrf2 and HO-1 Expression in ATI-like Cells

**Figure 5:**
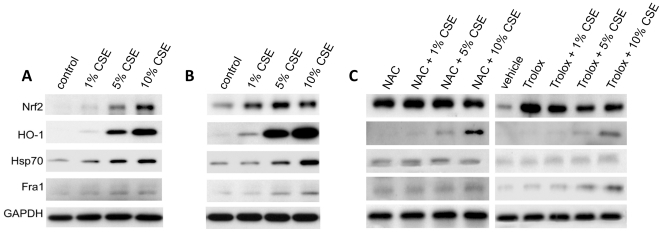
Protein expression after treatment with CSE and effect of co-treatment with NAC or Tolox. Representative time and concentration course expression of proteins in ATI-like cells exposed to CSE and after co-treatment with CSE and 5 µM NAC or CSE and 5 µM trolox. Proteins were measured by immunoblotting. A - Cells treated for 4 h; B - Cells treated for 24 h; C - Cells were treated with CSE and NAC or trolox for 24 h. Representative data each from one of three experiments are shown.

In order to confirm the observation of Nrf2 nuclear translocation in response to CSE in ATI-like cells as outlined above, we analyzed Nrf2 and other ROS-sensitive or apoptosis-related protein expression by western blotting. ATI-like cells were treated with 1%, 5%, and 10% CSE for 4 h and 24 h. We observed early statistically significant increases of Nrf2 (∼98 kDa), HO-1 (32 kDa), and Hsp70 (70 kDa) after application 5% and 10% CSE for 4 h (Figure 5A; Figure S2A). The highest expression was observed for HO-1. We did not find significant expression of Fra1 (43 kDa) after cell treatment for 4 h. We also analyzed protein expression after ATI-like cell treatment with CSE for 24 h (Figure 5B; Figures S2B–E). Nrf2 and HO-1 expressions were statistically significantly increased after application of all concentrations of CSE. Hsp70 expression was significantly increased after treatment with 5% and 10% CSE, and Fra1 after 10% CSE application. We found statistically significant higher expression of Nrf2, HO-1 and Hsp70 in ATI-like cells in comparison with ATII cells after cell treatment with 1% and 5% CSE (Figures S2, S3). Our results suggest a role for ROS as mediators of CSE induced stress response in ATI-like cells.

### NAC and Trolox Increase Nrf2 Expression but Reduce Hsp70

To determine whether application of NAC or trolox could attenuate the CSE-induced cellular stress and Nrf2 expression, we co-treated ATI-like cells with CSE and NAC, or CSE and trolox for 24 h. We found that 5 µM NAC or 5 µM trolox significantly induced expression of Nrf2, which suggests a protective mechanism of both antioxidant compounds against cell injury induced by CSE (Figure 5C; Figure S2B). Moreover, we found that co-treatment with CSE and NAC, or CSE and trolox for 24 h significantly attenuated HO-1 (Figure S2C) and Hsp70 expression (Figure S2D) induced by cell treatment with CSE. Collectively, our data suggest the protective effect of NAC or trolox on the CSE-induced cellular injury.

### CSE Induces a Similar Response in Differentiated ATII Cells but Requires a Higher Concentration

**Figure 6:**
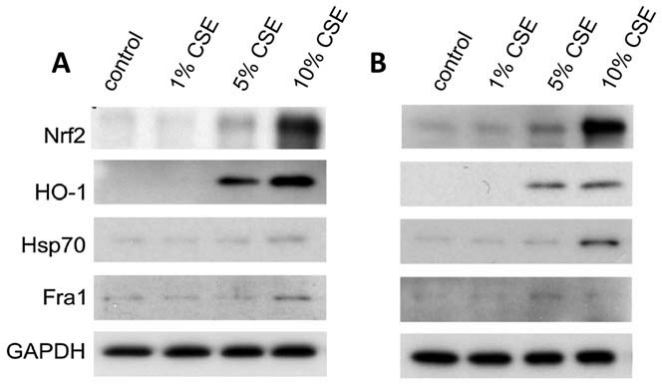
Time and concentration course expression of proteins in ATII cells exposed to CSE. A - for 4 h; B - for 24 h. Proteins were measured by immunoblotting. Representative data each from one of three experiments are shown.

ATII cells are known to be less sensitive to oxidant injury than type I cells *in vivo* and less sensitive to CSE (Figure S1). We therefore wanted to determine if this was likely just due to the dose or if the cellular response was totally different. Hence, we repeated the same studies with differentiated ATII cells. We found statistically significant increases in Nrf2, HO-1, and Hsp70 expression in ATII cells after 4 h and 24 h cell treatment with 5% and 10% CSE. We also analyzed expression of proteins in ATII cells treated with CSE for 4 h and 24 h (Figure 6A, B; Figure S3A, B). Fra1 expression was significantly increased after 4 h treatment with 10% CSE and after 24 h treatment with 5% CSE. We did not observe statistically significant increases in expression of all studied proteins after application of 1% CSE after 4 h or 24 h. Furthermore, Nrf2 expression was significantly induced after 5% and 10% CSE was added to ATII cells at 24 h and by all applied CSE concentrations in ATI-like cells at the same time point (Figure S2). Only Nrf2 expression after treatment with 10% CSE was statistically higher in ATII cells in comparison with ATI-like cells (Figure S3). However, this suggests a decrease in Nrf2 activation under very high oxidative stress [25], [26] in ATI-like cells. In summary, ATI-like cells are more sensitive especially after 24 h treatment with CSE than ATII cells but the general response was similar.

### Nrf2 Knockdown Sensitizes ATI-like Cells to Injury by CSE

**Figure 7:**
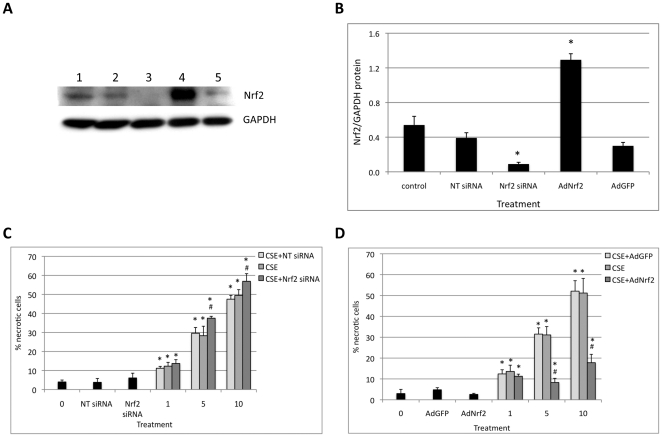
Effect of Nrf2 knockdown and overexpression in ATI-like cells exposed to CSE. A – Nrf2 expression (immunoblotting); Lane 1 – control; lane 2–300 nM NT siRNA; lane 3–300 nM Nrf2 siRNA; lane 4–200 MOI AdNrf2; lane 5–200 MOI AdGFP (see Materials and Methods); B - Quantitation of Nrf2 relative protein expression in ATI-like for experiments shown in A. * - Statistically significant increase in protein expression induced in comparison with negative control (*p*<0.05); C – Nrf2 knockdown sensitizes cells to CSE. Cells were transfected with Nrf2 siRNA for 24 h followed by exposure to CSE for 24 h and toxicity was measured by propidium iodide and Hoechst 33342 double staining. D - Nrf2 overexpression protects cells exposed to CSE. Cells were infected with AdNrf2 for 24 h, exposed to CSE for 24 h and necrotic cells were detected by Hoechst 33342 and propidium iodide double staining. * - Statistically significant increase in percentage of necrotic cells induced by CSE in comparison with control. # - Statistically significant increase for C or decrease in D in ATI-like necrotic cells after transfection with Nrf2 siRNA or infection with AdNrf2, respectively followed by CSE in comparison with CSE alone. Data represent results from three independent experiments (*p*<0.05).

We used Nrf2 overexpression or knockdown to provide a direct link between CSE and Nrf2. We were able to knockdown Nrf2 in ATI-like cells (Figure 7A, B). We found that cell transfection with Nrf2 siRNA followed by exposure to 5% or 10% CSE induced a significant increase in the percentage of necrotic cells in comparison with CSE alone (Figure 7C). We did not observe a statistically significant increase for 1% CSE. These results suggest that Nrf2 knockdown sensitizes cells to CSE, which provides a direct link between this gene and cell death.

### Nrf2 Overexpression Protects ATI-like Cells Against Injury by CSE

To complement studies described above, we used Nrf2 overexpression in ATI-like cells. We found that cell infection with adenovirus Nrf2 (AdNrf2) followed by treatment with 5% or 10% CSE significantly decreased the number of necrotic cells in comparison with CSE alone (Figure 7D). We did not observe a significant decrease for 1% CSE. This experiment confirms our data on the protective role of Nrf2 in cells exposed to CSE.

### NAC and Trolox Protect Cells with Knocked Down Nrf2

**Figure 8:**
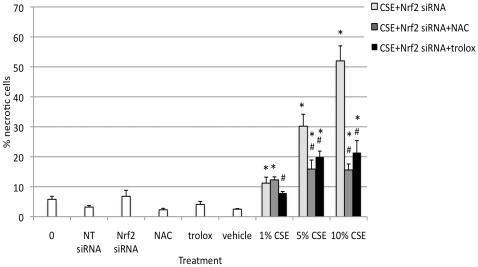
NAC and trolox protect ATI-like cells with knocked down Nrf2 against injury induced by CSE. Nrf2 was knocked down and cells were exposed for 24 h to CSE and NAC or trolox and toxicity was measured by propidium iodide and Hoechst 33342 double staining. * - Statistically significant increase in percentage of necrotic cells in comparison with control, NAC or trolox, respectively. # - Statistically significant decrease of ATI-like necrotic cells after co-treatment with CSE and NAC or CSE and trolox in comparison with cell exposure to CSE. Data represent results from three independent experiments (*p*<0.05).

We knocked down Nrf2 in ATI-like cells followed by treatment with CSE and NAC or trolox to verify the protective role of NAC and trolox against CSE. We found a significantly lower percentage of necrotic ATI-like cells after treatment with 5% CSE or 10% CSE and NAC or trolox (Figure 8) in comparison with CSE alone. Trolox but not NAC also protected cells treated with 1% CSE. These results suggest that both antioxidant compounds may also protect cells against injury induced by CSE through other pathways than Nrf2 and may directly neutralize the oxidant in CSE or the initial ROS generated in cells exposed to CSE.

## Discussion

Oxidative stress induced by CS plays a key role in the pathogenesis of many pulmonary diseases [27]. However, the toxicity has been variably attributed to apoptosis and necrosis. We found that ATI-like cells are more sensitive to CS than differentiated ATII cells. Since human type I cells have not been isolated and cultured, we used ATI-like cells, which are type II cells cultured to transdifferentiate into type I-like cells *in vitro* and express a similar but slightly different gene profile to isolated type I cells [23], [28]. This study confirmed our hypothesis that Nrf2 plays a direct role in preventing injury to alveolar epithelial cells by CSE. Nrf2 overexpression protected cells and its knockdown sensitized cells to CSE-induced cell death. Moreover, we also found that NAC and trolox significantly decreased the percentage of necrotic cells observed after treatment with CSE. Furthermore, our results suggest that both antioxidant compounds may also protect ATI-like cells against CSE through pathways other than Nrf2.

We observed induction of both apoptosis and necrosis in a time- and concentration-dependent manner after cell treatment with CSE. We found a higher percentage of concentration-dependent induction of apoptosis than necrosis after 4 h ATI-like cell treatment with CSE and more necrosis after 24 h cell treatment with CSE. First, our results are in agreement with observations that lung cell apoptosis tends to occur when CSE is applied for a shorter time or at lower concentrations, whereas cells go into necrosis when the CSE is given for a longer time or at higher concentrations [2], [29], [30], [31]. Second, we found that CSE decreased mitochondrial membrane potential in ATI-like cells after 24 h (Data S1; Figure S4A, B, C) and this may suggest that dysfunctional mitochondria may enhance susceptibility to cell death [32].

Moreover, a consequence of ROS production and the increased oxidative stress response associated with CS is lipid peroxidation. A specific end product of lipid peroxidation is 4-HNE, which can react with DNA and proteins to generate various adducts that can induce cellular response, including apoptosis [33]. We observed 4-HNE positive ATI-like cells and intense staining in the cytoplasm and nucleus. It was also reported that CSE increased 4-HNE levels in human lung fibroblasts, and this staining was observed in the cytoplasm, nucleus and in the nuclear membrane [33]. Thus lipid peroxidation associated with increased ROS production may be contributing to the CS-induced ATI-like cell apoptosis observed in our studies.

Upon exposure to high concentrations of ROS found in CS, lung tissue responds by activating the transcription of a battery of genes that either aid in the detoxification of ROS or induce the repair of the resulting intracellular oxidative damage [1]. We observed significant Nrf2 expression in our studies with ATI-like and ATII cells after exposure to CSE. Moreover, we found that after ATI-like cell exposure to CSE, Nrf2 translocates to the nucleus coinciding with significant expression of HO-1. We observed the higher nuclear translocation of Nrf2 in ATI-like cells treated with CSE for 4 h than for 24 h. It was suggested that at this earlier time point, the oxidants in CSE oxidize the thiol group, resulting in dissociation of Nrf2 from Keap1 and translocation of Nrf2 into the nucleus. At 24 h CSE exposure, aldehydes present in CSE or formed by lipid peroxidation form protein carbonyl adducts with sulfhydryl (SH) groups of Nrf2/Keap1, thereby leading to modulation of these groups [34].

We observed significant increases in Nrf2 and HO-1 expression after ATI-like and ATII cell treatment with CSE for 4 h and 24 h. The upregulation of HO-1 likely has a protective role to counteract oxidative stress caused by CSE [27]. We also found significant expression of Hsp70 and Fra1 after ATI-like and ATII cell exposure to CSE. Increased expression of Hsp70 may represent a protective mechanism against injury [35].

We also knocked down and overexpressed Nrf2 to analyze its direct role in ATI-like cells exposed to CSE. We found that Nrf2 overexpression protects cells and its knockdown sensitizes cells to CSE-induced cell death. These results suggest the potential importance of Nrf2 targeting to protect cells against CS. Furthermore, it has already been suggested that Nrf2 may be a potential valuable therapeutic target in pulmonary emphysema [36].

NAC completely abolished the cytotoxicity of CSE in A549 cells [30], attenuated ROS production and apoptosis induced by CSE in three different human lung fibroblast strains [33] and significantly decreased necrosis in our studies after co-treatment with CSE and NAC in comparison with CSE alone. Moreover, Ji et al. [37] found that NAC elevated Nrf2 expression significantly in rat lungs. NAC protected against oxidative stress through acting on Nrf2/glutathione reductase/GSH pathway, by which NAC elevated the biosynthesis of protective GSH to repair and reconstitute the defense system. This may explain the higher expression of Nrf2 observed in our studies in ATI-like cells after application of NAC. We observed significant attenuation of HO-1 expression after ATI-like cell co-treatment with CSE and NAC that may suggest that NAC acts as a GSH precursor to raise intracellular GSH level or as a thiol decoy, or both [38]. However, we did not observe the decrease in Fra1 expression after CSE and NAC cell co-treatment. This suggests that oxidative stress plays only a minimal role in mediating CSE-stimulated *fra1* induction. However, we observed significant decreases in expression of Hsp70 after co-treatment with CSE and NAC in comparison with application of CSE. Vayssier-Taussat [35] reported that pretreatment of monocytes and endothelial cells with NAC before introduction of CSE completely abolished CSE-mediated induction of Hsp70 expression by first, decreasing the stress responses induced by CSE and second, by inhibiting the intracellular GSH depletion secondary to CSE exposure thus preventing lipid peroxidation.

Trolox prevents an increase in hydroxyl radical mediated cytotoxcity, lipid peroxidation, and cell death [21], [39], [40]. We found less necrosis after ATI-like cell co-treatment with CSE and trolox than with CSE alone. We also observed a significant increase of Nrf2 after ATI-like cell treatment with trolox. This compound, possibly through the reduction of ROS, prevents the decrease in GSH levels [41] and this may also explain the protective effect of trolox in cells with knocked down Nrf2. We also observed that co-treatment with CSE and trolox significantly decreased expression of HO-1 in comparison to treatment with CSE alone. HO-1 expression was attenuated by co-treatment with CSE and trolox or co-treatment with CSE and NAC. We did not observe HO-1 induction by NAC and trolox alone. HO-1 activation by Nrf2 represents a late event in the antioxidant response [42]. We also observed a lower expression of all stress related proteins in ATII cells treated with CSE as compared to ATI-like cells.

It was already reported that oxidative stress has been implicated in the pathogenesis of pulmonary emphysema and CS is a major causative factor in this disease's development [13]. Studies using Nrf2-knockout mice identified an extensive range of protective roles for Nrf2 against pathogenesis of this disease [36], [43]. Iizuka et al. [44] reported that in Nrf2^−^/^−^ mice, emphysema was first observed at 8 weeks and exacerbated by 16 weeks following CS-exposure, whereas no pathological abnormalities were observed in wild-type mice. Moreover, emphysema in Nrf2-deficient mice exposed to CS for 6 months was associated with more pronounced bronchoalveolar inflammation, oxidative stress in alveolar cells and an increased number of apoptotic alveolar septal cells as compared with wild-type mice. Furthermore, the expression of nearly 50 Nrf2-dependent antioxidant and cytoprotective genes in the lung were identified by microarray analysis that may work in concert to counteract CS-induced oxidative stress and inflammation [43]. It is important to note that the potent Nrf2 activator, CDDO-Im, significantly reduced lung oxidative stress, alveolar cell apoptosis, alveolar destruction, and pulmonary hypertension in wild-type mice caused by chronic exposure to CS. This protection from CS-induced emphysema depended on Nrf2, as Nrf2^−^/^−^ mice failed to show significant reduction in alveolar cell apoptosis and alveolar destruction after treatment with this compound. These results suggest that targeting the Nrf2 pathway may represent an important approach for prophylaxis against COPD [45]. Our results using human primary alveolar epithelial cells improve our understanding of the protective role of Nrf2 in CSE-induced injury. It is important to note that Nrf2 levels are lower in lung tissue and alveolar macrophages from smoking-related pulmonary emphysema [10], [13], [46]. Therefore antioxidant compounds targeting Nrf2 may represent a potential therapeutic strategy in this disease.

In summary, our study shows sensitivity of human alveolar epithelial cells to CSE and the concept that Nrf2 activation is a major cellular defense against CSE-induced oxidative stress. Our results are in agreement with observations that ROS production initiates the activation of Nrf2 and activated Nrf2 is recruited to the promoter activity and the expression of *HO-1* mRNA [47]. The susceptibility of smokers to CS-dependent diseases may be based on the defense mechanism orchestrated by Nrf2. Therefore antioxidant agents that modulate Nrf2 would be expected to restore antioxidant and detoxifying enzymes by upregulation of phase 2 genes, counteract CS toxicity and to prevent of CS-related lung injury. Our results suggest that both NAC and trolox can prevent the cytotoxicity to human alveolar epithelial cells due to CSE.

## Materials and Methods

### Isolation and Culture of ATII Cells and ATI-like Cells

Deidentified human lungs not suitable for transplantation were donated for medical research from the National Disease Research Interchange (Philadelphia, PA) and the International Institute for the Advancement of Medicine (Edison, NJ). In the present study we selected donors with reasonable lung function with a PaO_2_/FIO_2_ ratio of >250, a clinical history and x-ray that did not indicate infection, and limited time on a ventilator. We know the age, gender, race, smoking history, cause of death, very brief medical history, and medications at the time of death. The Committee for the Protection of Human Subjects at National Jewish Health approved this research. In this study all the ATII cells were isolated from 6 non-smokers.

The ATII cell isolation method has been published previously [24]. Briefly, the right middle lobe was perfused, lavaged, and then instilled with elastase (12.9 U/ml; Roche Diagnostics, Indianapolis, IN). The lung was minced and subsequently the cells were filtrated and purified by centrifugation on a density gradient made of Optiprep (Accurate Chemical Scientific Corp., Westbury, NY) and by negative selection with CD14-coated magnetic beads (Dynal Biotech ASA, Oslo, Norway) and binding to IgG-coated (Sigma Chemicals Inc., St. Louis, MO) dishes. The cells were counted and cell purity was estimated by staining for cytokeratin CAM 5.2 (Dako, Carpinteria, CA). The purity of ATII cells was ∼80% before plating and over 95% after adherence in culture [23].

The isolated ATII cells were resuspended in DMEM supplemented with 10% fetal bovine serum (FBS; Thermo Scientific HyClone, Franklin, MA), 2 mM glutamine (Thermo Scientific HyClone, Franklin, MA), 2.5 µg/ml amphotericin B (Mediatech Inc., Manassas, VA), 100 µg/ml streptomycin (Thermo Scientific HyClone, Franklin, MA), 100 U/ml penicillin (Thermo Scientific, Franklin, MA), and 10 µg/ml gentamicin (Sigma Chemicals Inc., St. Louis, MO). To maintain their differentiated state, ATII cells were plated for 2 days on millicell inserts (Millipore Corp., Bedford, MA) that had been previously coated with a mixture of 20% Engelbreth-Holm-Swarm tumor matrix (BD Biosciences, San Jose, CA) and 80% rat-tail collagen (RTC) in DMEM with 10% FBS and then cultured for two days with 1% charcoal-stripped FBS along with 10 ng/ml keratinocyte growth factor (R&D Systems Inc., Minneapolis, MN), and for an additional two days with 10 ng/ml keratinocyte growth factor, 0.1 mM isobutylmethylxanthine, 0.1 mM 8-Br-cAMP, and 10 nM dexamethasone in addition to glutamine, amphotericin B, streptomycin, penicillin, and gentamicin as mentioned above [24], [48].

To transdifferentiate ATII cells into ATI-like cells, type II cells were cultured on RTC-coated tissue culture plates, glass coverslips or Lab-Tek chamber slides (Nalge Nunc International, Rochester, NY). Cells were plated for two days in DMEM with 10% FBS and subsequently cultured for four days in DMEM with 5% FBS [23].

### Preparation of CSE

The CSE was prepared as previously described [49] with a slight modification. Briefly, the smoke of one 3R4F cigarette without filter (Kentucky Tobacco Research & Development Center, Lexington, KY) containing 9.5 mg tar and 0.72 mg nicotine [50] was drawn into 12.5 ml DMEM with peristaltic pump (Manostat 72-310-000; Barnant Company, Barrington, IL). The pump was set at an optimum speed to allow one cigarette to burn in approximately 15 minutes and resulting solution was considered 100% CSE. This solution was filtered through a 0.22 µm pore acrodisc syringe filter and applied immediately to the cell cultures. In our studies ATI-like cells and ATII cells were treated with 1%, 5%, and 10% CSE for 4 h and 24 h. Based on the published assumption that a human generates 350 ml smoke with each cigarette and has a blood volume of 6 liters [51], [52], [53] we calculated that in our studies 1% CSE is equal to 5 cigarettes/day, 5% CSE is equal to 25 cigarettes/day and 10% CSE is equal to 50 cigarettes/day which corresponds to the realistic situation of human daily exposure to CS.

### Chemical Compounds

Trolox (CAS 53188-07-1) was purchased from Calbiochem - EMD Biosciences, Inc., La Jolla, CA and NAC (CAS 616-91-1) from Sigma Chemicals Inc., St. Louis, MO. Trolox was dissolved in ethanol and the final concentration of ethanol in the cultures did not exceed 0.04% (v/v), and NAC was dissolved in PBS.

### Identification of Apoptosis and Necrosis

#### a.) Acridine Orange and Ethidium Bromide Double Staining

Double staining with acridine orange and ethidium bromide (both from Sigma Chemicals Inc., St. Louis, MO) allows to distinguish between: (i) living cells (green nucleus with red-orange cytoplasm); (ii) early apoptosis stage (cell membrane still continuous but chromatin condensation with an irregular green nucleus is visible); (iii) late apoptosis (so called ‘secondary necrosis’ or ‘apoptotic necrosis’- orange nuclei, fragmentation or condensation of chromatin is still observed); and (iv) necrosis (uniform orange-coloured cell nuclei) [54]. 300 cells were immediately analyzed and scored for these four stages by fluorescence microscopy (Zeiss Axiovert 200 M, Carl Zeiss, Germany) following the addition of 4 µg/ml acridine orange and 4 µg/ml ethidium bromide in three independent experiments.

#### b.) Hoechst 33342 and Propidium Iodide Double Staining

Hoechst 33342 and propidium iodide (both from Sigma Chemicals Inc., St. Louis, MO) at 0.01 mg/ml and 0.001 mg/ml respectively, were used to distinguish between live and necrotic cells after treatment with CSE only, CSE and NAC or CSE and trolox. 300 cells were analyzed by fluorescence microscopy (Zeiss Axioskop 2, Carl Zeiss, Germany) in three independent experiments.

#### c.) TUNEL Assay

The TdT-mediated dUTP Nick-End Labeling (TUNEL; Promega, Madison, WI) assay was used to compare the ability of CSE to induce apoptosis as previously described [55]. Briefly, ATI-like cells were fixed in 4% paraformaldehyde (Electron Microscopy Sciences, Hatfield, PA) in PBS and permeabilized with 0.2% triton X-100 (Sigma Chemicals Inc., St. Louis, MO). Then, slides with cells were incubated for 1 h at 37°C in a humid chamber in the presence of terminal deoxynucleotidyl transferase (TdT). In the negative control no TdT was added whereas in the positive control 10 U/ml DNase (Promega, Madison, WI) was used (data not shown), respectively according to manufacturer's recommendations. Cells were mounted with Vectashield medium containing DAPI (Vector Laboratories, Burlingame, CA) and analyzed by fluorescence microscopy (Zeiss Axioskop 2, Germany). The percentage of TUNEL-positive apoptotic ATI-like cells labeled with fluorescein (fluorescein-dUTP-labelled DNA) was calculated per 10 high-power fields (magnification 10×40) [56].

#### d.) Detection of Caspase 3 and Caspase 7 Activity

Concentration- and time-dependent caspase 3 and caspase 7 activities were analyzed in ATI-like cells treated with CSE with a commercial Caspase-Glo 3/7 Assay kit (Promega, Madison, WI). ATI-like cells were treated with 1%, 5%, and 10% CSE for 4 h and 24 h, and both attached and detached cells were collected for this assay. We used 20,000 cells equilibrated to room temperature for this experiment. Caspase-Glo 3/7 reagent was added for 1 h of incubation and then the luminescence signal was measured by a luminometer (Synergy HT, BioTek) according to the manufacturer's protocol.

### Immunocytofluorescence

To detect Nrf2 translocation induced by CSE, NAC or trolox ATI-like cells cultured on cover slips were fixed in 100% methanol and washed in PBS. After blocking with 3% normal donkey serum (Jackson ImmunoResearch; West Grove, PA) in PBS, the cells were incubated with rabbit anti-Nrf2 (H-300) antibody (Santa Cruz Biotechnology Inc., Santa Cruz, CA). The secondary antibody, Alexa Fluor 594 anti-rabbit IgG (Invitrogen Corp., Carlsband, CA) was applied with the cells for 1 h, and cells were mounted with Vectashield medium containing DAPI.

To analyze lipid peroxidation generated by CSE, ATI-like cells were fixed in 4% paraformaldehyde, washed in PBS, permeabilized 10 min in 0.2% Triton X-100, washed in PBS, and subsequently blocked with 3% normal donkey serum in PBS. Cells were incubated with a mouse monoclonal 4-hydroxy-2-nonenal antibody (4-HNE; OXIS International Inc., Foster City, CA). Subsequently, the secondary antibody, Alexa Fluor 488 anti-mouse IgG (Invitrogen Corp., Carlsband, CA) was used, and cells were mounted with Vectashield medium containing DAPI.

### Western Blotting

ATI-like cells and ATII cells were treated with CSE for 4 h or 24 h. We also applied co-treatment of CSE and NAC or CSE and trolox for 24 h in ATI-like cells. We collected both attached and detached cells for this assay. Expression of protein was measured by western blotting according to protocol described previously [24]. Briefly, polyacrylamide gradient gels (8–16%; Invitrogen Corp., Carlsband, CA) were run in tris glycine buffer to separate the proteins. Protein loading was normalized to mouse anti-glyceraldehyde-3-phosphate dehydrogenase (anti-GAPDH; 40 kDa) purchased from Abcam (Cambridge, MA). Mouse anti-HO-1 (Hsp32) was purchased from Assay Designs (Ann Arbor, MI), and the following antibodies were purchased from Santa Cruz Biotechnology (Santa Cruz, CA): mouse anti-Hsp70 (4E7), rabbit anti-Nrf2 (H-300), and rabbit anti-Fra1 (R-20). Horseradish peroxidase (HRP)-conjugated AffiniPure donkey anti-rabbit immunoglobulin (Ig) G and HRP-conjugated AffiniPure donkey anti-mouse IgG were purchased from Jackson ImmunoResearch (West Grove, PA). The blots were then developed using an enhanced chemiluminesence western blotting kit according to the manufacturer's instructions (Amersham Pharmacia Biotech, Piscataway, NJ). Images were quantitated using NIH Image 1.62 software.

### Nrf2 Overexpression

AdNrf2 and adenovirus green fluorescent protein (AdGFP) were obtained from Dr. Timothy H. Murphy [57]. ATI-like cells were infected by adenovirus diluted to a multiplicity of infection (MOI) of 200 in PBS. Cells were allowed to express transgenes for 24 h before treatment with CSE. All infected cell cultures were examined for adequate infection efficiency (86%) as assessed by GFP fluorescence and by western blotting.

### Nrf2 Knockdown

Nrf2 siRNA (small inhibitory RNA) duplex showing maximum knockdown in A549 cells (sense: 5′ CAGCAGAACUGUACCUGUUUU 3′; antisense: 3′ UUGUCGUCUUGACAUGGACAA 5′) [58] was purchased from Dharmacon Research, Inc (Lafayette, CO). To confirm the specificity of the inhibition, the control, nontargeting siRNA was used as negative control (sense: 5′ UAGCGACUAAACACAUCAAUU 3; antisense 3′ UUAUCGCUGAUUUGUGUAGUU 5′) [58]. Cells were transfected with 300 nM of siRNA duplexes by using GenomONE HVJ Envelope Vector Kit (Cosmo Bio CO. Ltd. Carlsbad, CA) according to the manufacturer's recommendations. After 24 h, cells were treated with CSE as described above. Knockdown of the target gene was quantified by western blotting.

We also knocked down Nrf2 (24 h) followed by treatment with CSE and NAC or trolox for 24 h as described above. This approach was used to verify the direct link between Nrf2, CSE and NAC or trolox.

### Statistical analysis

One-way ANOVA by GraphPad Prism 4 was used to evaluate statistical differences among experimental groups. A Dunnett's test was applied and a value of *p*<0.05 was considered significant. Data are shown here as the mean ± SEM from three independent experiments.

## Supporting Information

Figure S1 **ATII cells are more resistant to CSE than ATI-like cells as detected by Hoechst 33342 and propidium iodide double staining.** A - ATI-like cells were analyzed after 4 h and 24 h treatment with CSE; B - ATII cells were treated with CSE for 4 h and 24 h. * - Statistically significant increase in percentage of necrotic cells induced by CSE in comparison with control. # - Statistically significant increase of ATI-like necrotic cells in comparison with necrotic ATII cells after treatment with CSE. Data represent results from three independent experiments (*p*<0.05).
(TIF)

Figure S2 **Time and concentration course expression of proteins in ATI-like cells.** ATI-like cells were treated with CSE or cotreated with CSE and NAC or CSE and trolox. Proteins were measured by immunoblotting using NIH Image 1.62 software. A - Quantitation of Nrf2, HO-1, Hsp70 and Fra1 relative protein expression after ATI-like cell treatment with CSE for 4 h for experiments shown in Figure 5A. Quantitation of Nrf2 (B), HO-1 (C), Hsp70 (D) and Fra1 (E) protein levels relative to GAPDH were measured after ATI-like cell treatment with CSE for 24 h, cotreatment for 24 h with CSE and NAC or CSE and trolox for experiments shown in Figure 5B, C. * - Statistically significant increase in protein expression induced by CSE in comparison with negative control. # - Statistically significant decrease in HO-1 and Hsp70 protein expression after ATI-like cell cotreatment with CSE and NAC or CSE and trolox in comparison with CSE. § - Statistically significant increase in protein expression in ATI-like cells in comparison with results for ATII cells presented in Figure S3. All experiments were repeated three times (*p*<0.05).
(TIF)

Figure S3 **Time and concentration course expression of proteins in ATII cells.** ATII cells were treated with CSE for 4 h and 24 h. Proteins were measured by immunoblotting using NIH Image 1.62 software. A - Quantitation of Nrf2, HO-1, Hsp70 and Fra1 relative protein expression after ATII cell treatment with CSE for 4 h for experiments shown in Figure 6A. B - Quantitation of Nrf2, HO-1, Hsp70 and Fra1 relative protein expression after ATII cell treatment with CSE for 24 h for experiments shown in Figure 6B. * - Statistically significant increase in protein expression induced by CSE in comparison with negative control. § - Statistically significant differences in protein expression in comparison with results for ATI-like cells presented in Figure S2. All experiments were repeated three times (*p*<0.05).
(TIF)

Figure S4 **CSE reduces the mitochondrial membrane potential in ATI-like cells.** A – In control cells a red multimeric form of dye accumulates in healthy mitochondria as detected by the DePsipher^TM^ assay. The green monomeric form of the dye in cytoplasm is observed when mitochondrial membrane collapses in apoptotic cells after incubation with 3 µM valinomycin for 24 h (B) or after exposure to 5% CSE for 24 h (C). Representative data each from one of three experiments are shown.
(TIFF)

Data S1 **Supplementary data.**
(DOC)
