# Supplementary figures and images for: Human Alveolar Epithelial Cell Injury Induced by Cigarette Smoke
Source: PLoS One. 2011 Dec 7;6(12):e26059. doi: 10.1371/journal.pone.0026059 (PMC3233536; doi:10.1371/journal.pone.0026059)

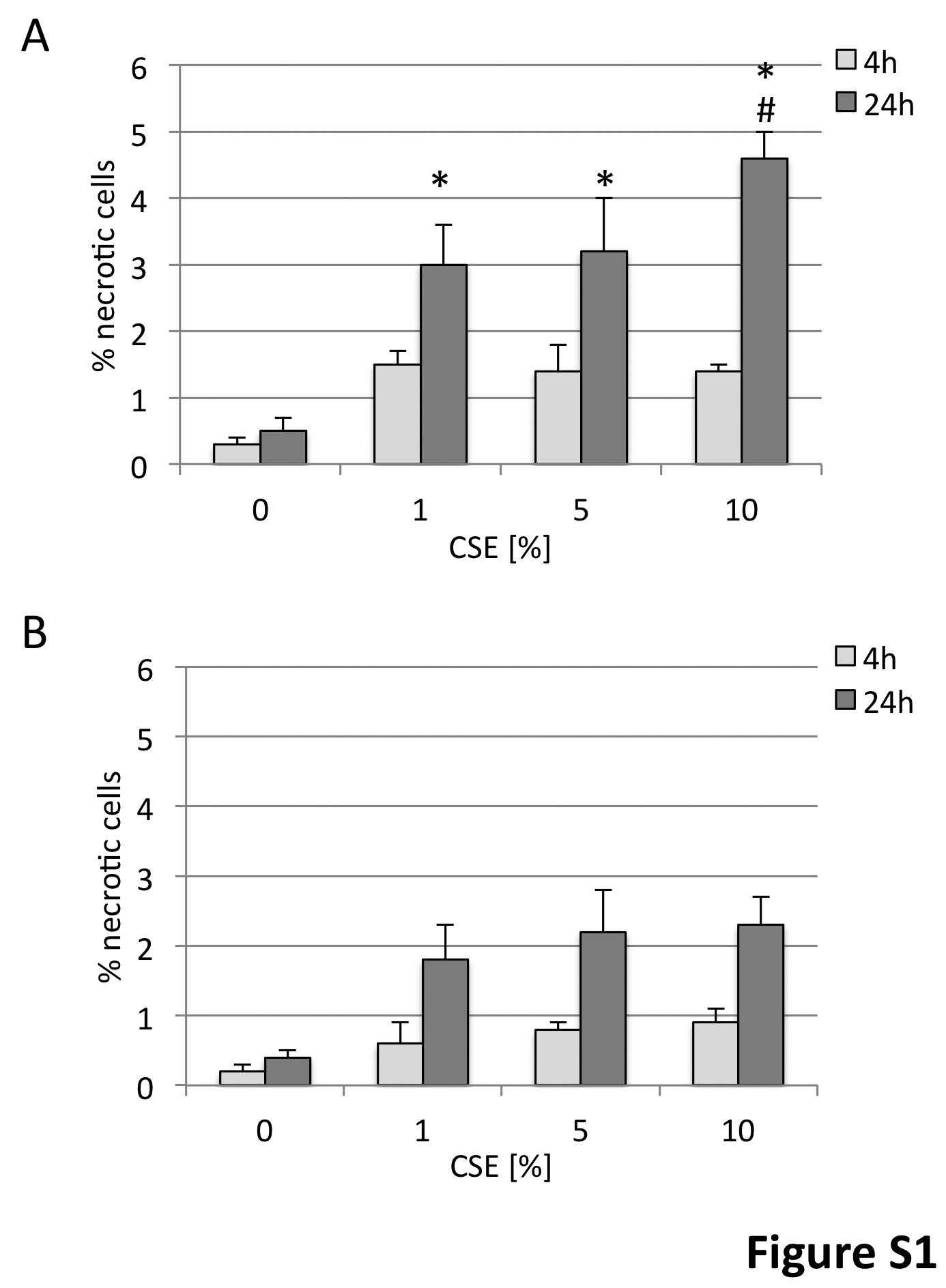

Supplement: Figure S1 — ATII cells are more resistant to CSE than ATI-like cells as detected by Hoechst 33342 and propidium iodide double staining. A - ATI-like cells were analyzed after 4 h and 24 h treatment with CSE; B - ATII cells were treated with CSE for 4 h and 24 h. * - Statistically significant increase in percentage of necrotic cells induced by CSE in comparison with control. # - Statistically significant increase of ATI-like necrotic cells in comparison with necrotic ATII cells after treatment with CSE. Data represent results from three independent experiments (p<0.05). (TIF) [file pone.0026059.s001.tif]

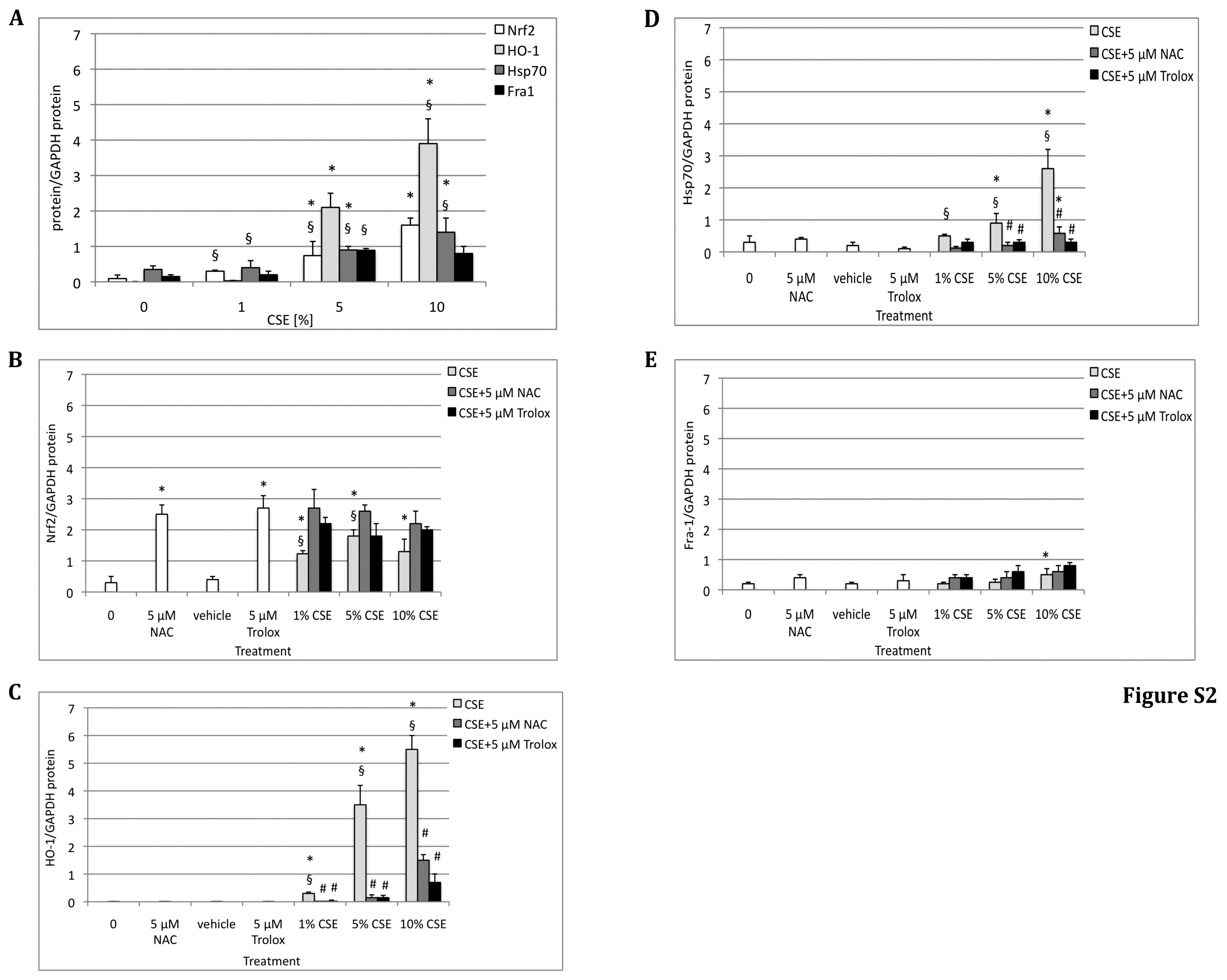

Supplement: Figure S2 — Time and concentration course expression of proteins in ATI-like cells. ATI-like cells were treated with CSE or cotreated with CSE and NAC or CSE and trolox. Proteins were measured by immunoblotting using NIH Image 1.62 software. A - Quantitation of Nrf2, HO-1, Hsp70 and Fra1 relative protein expression after ATI-like cell treatment with CSE for 4 h for experiments shown in Figure 5A. Quantitation of Nrf2 (B), HO-1 (C), Hsp70 (D) and Fra1 (E) protein levels relative to GAPDH were measured after ATI-like cell treatment with CSE for 24 h, cotreatment for 24 h with CSE and NAC or CSE and trolox for experiments shown in Figure 5B, C. * - Statistically significant increase in protein expression induced by CSE in comparison with negative control. # - Statistically significant decrease in HO-1 and Hsp70 protein expression after ATI-like cell cotreatment with CSE and NAC or CSE and trolox in comparison with CSE. § - Statistically significant increase in protein expression in ATI-like cells in comparison with results for ATII cells presented in Figure S3. All experiments were repeated three times (p<0.05). (TIF) [file pone.0026059.s002.tif]

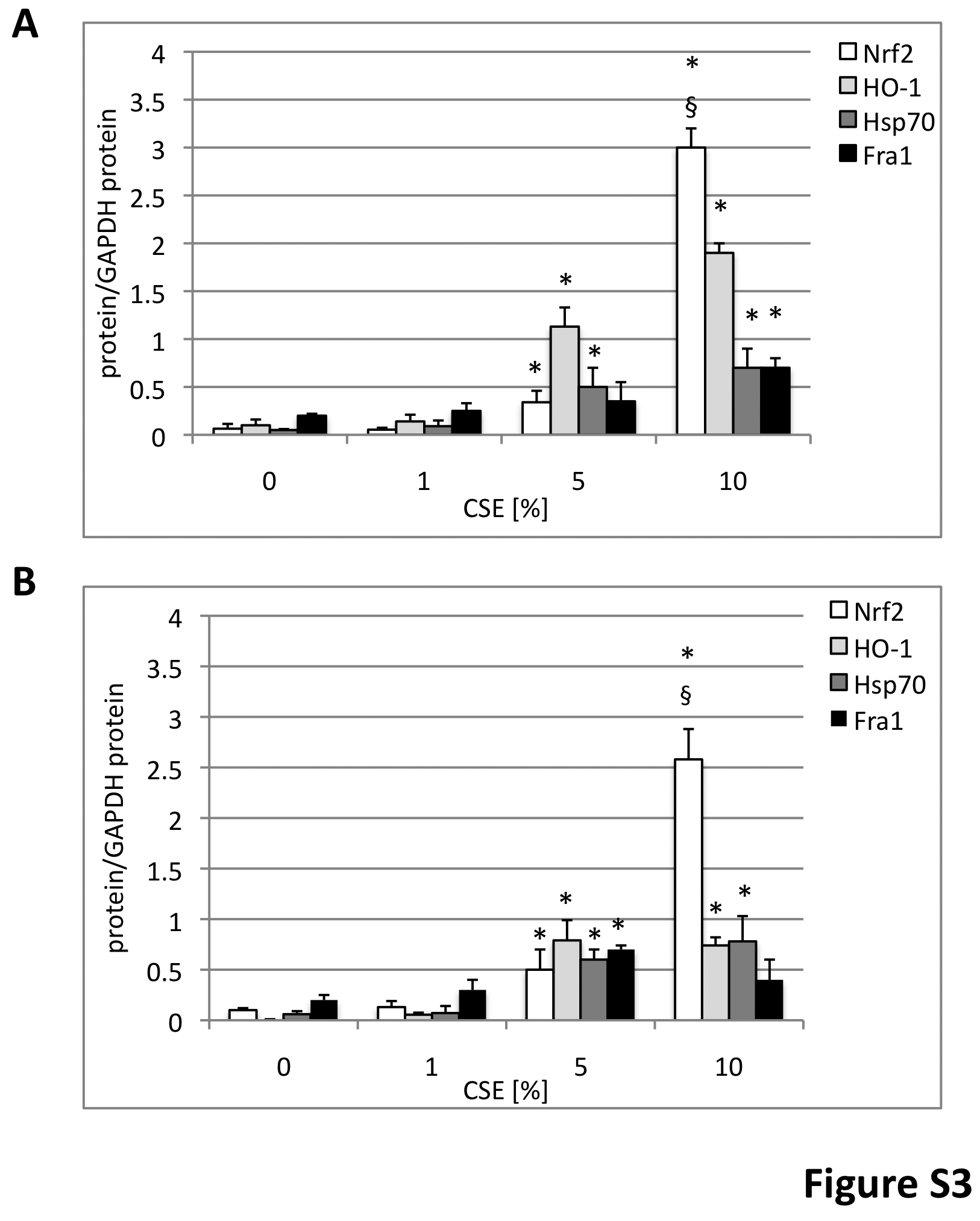

Supplement: Figure S3 — Time and concentration course expression of proteins in ATII cells. ATII cells were treated with CSE for 4 h and 24 h. Proteins were measured by immunoblotting using NIH Image 1.62 software. A - Quantitation of Nrf2, HO-1, Hsp70 and Fra1 relative protein expression after ATII cell treatment with CSE for 4 h for experiments shown in Figure 6A. B - Quantitation of Nrf2, HO-1, Hsp70 and Fra1 relative protein expression after ATII cell treatment with CSE for 24 h for experiments shown in Figure 6B. * - Statistically significant increase in protein expression induced by CSE in comparison with negative control. § - Statistically significant differences in protein expression in comparison with results for ATI-like cells presented in Figure S2. All experiments were repeated three times (p<0.05). (TIF) [file pone.0026059.s003.tif]

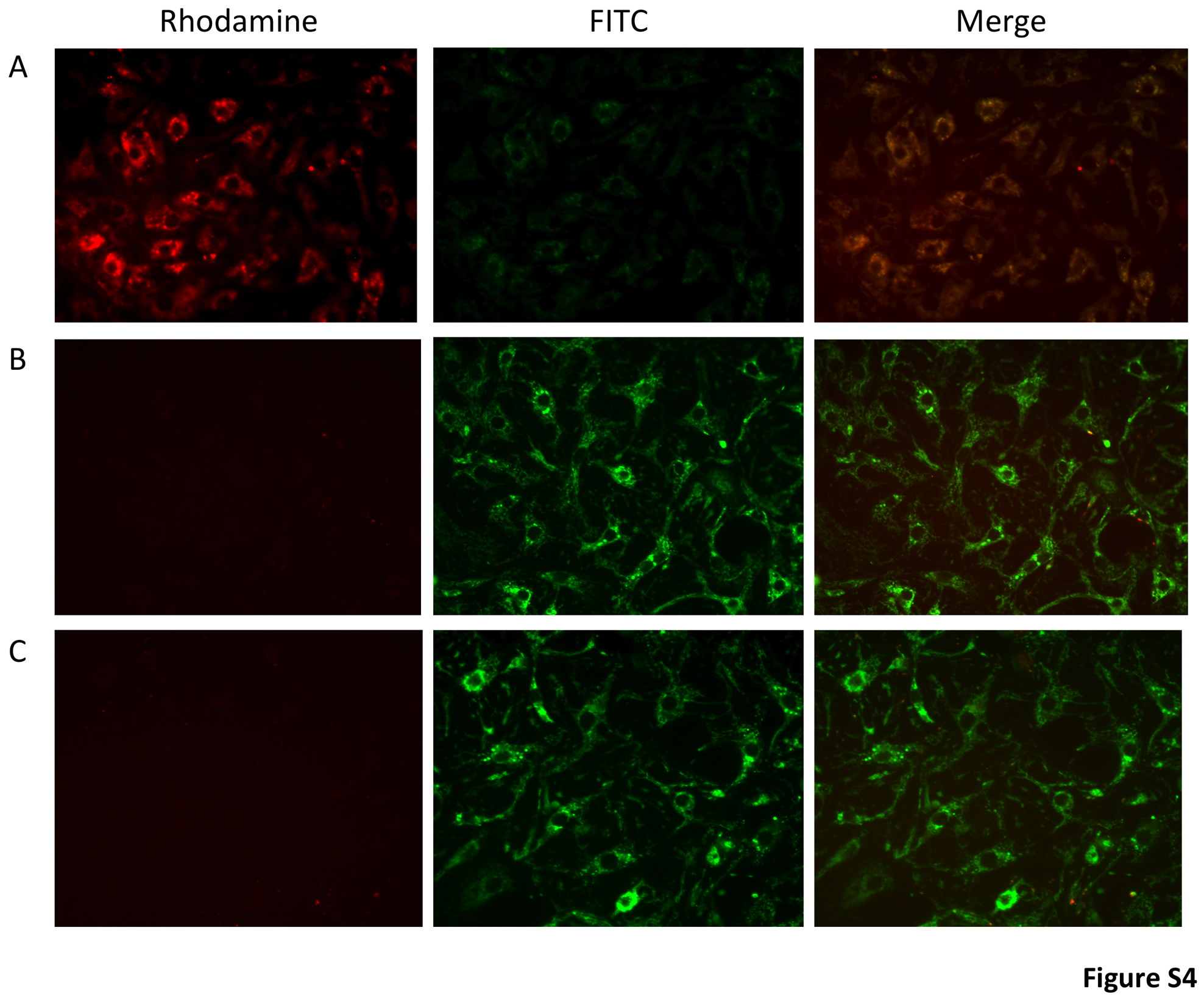

Supplement: Figure S4 — CSE reduces the mitochondrial membrane potential in ATI-like cells. A – In control cells a red multimeric form of dye accumulates in healthy mitochondria as detected by the DePsipherTM assay. The green monomeric form of the dye in cytoplasm is observed when mitochondrial membrane collapses in apoptotic cells after incubation with 3 µM valinomycin for 24 h (B) or after exposure to 5% CSE for 24 h (C). Representative data each from one of three experiments are shown. (TIFF) [file pone.0026059.s004.tiff]
