# Supplementary material for: Human Alveolar Epithelial Cell Injury Induced by Cigarette Smoke
Source: PLoS One. 2011 Dec 7;6(12):e26059. doi: 10.1371/journal.pone.0026059 (PMC3233536; doi:10.1371/journal.pone.0026059)
Supplement: Data S1 — Supplementary data. (DOC) [file pone.0026059.s005.doc]

**Supplementary Data**

**Human alveolar epithelial cell injury induced by cigarette smoke**

Beata Kosmider, Elise M. Messier, Hong Wei Chu, Robert J. Mason

Department of Medicine, National Jewish Health, 1400 Jackson Street, Denver, Colorado 80206

Correspondence:

Beata Kosmider, Ph.D.

Department of Medicine
National Jewish Health
1400 Jackson Street
Denver, CO 80206

E-mail: [KosmiderB@NJHealth.org](mailto:KosmiderB@NJHealth.org)

Phone: +1 303 270 2036

Fax: + 1 303 270 2353

**Supplementary Results**

CSE Disrupts the Mitochondrial Membrane Potential

We analyzed the effect of CSE on mitochondrial potential in ATI-like cells using the DePsipher^TM^ kit. We treated these cells with 5% CSE for 24 h, and we found a decrease of mitochondrial potential (Figure S4C).

**Supplementary Materials and Methods**

MTT Assay

The MTT assay [3(4,5-dimethylthiazol-2-yl) 2,5-diphenyltetrazolium bromide] was used as previously described [1] to determine non-toxic concentrations of NAC and trolox used in this study. ATI-like cells were cultured on 96-well plates and treated with these compounds for 24 h. Briefly, 200 μl of fresh DMEM and 50 μl MTT reagent (2 mg/ml) were added to the cells and incubated for 1 h at 37^o^C. DMEM was then removed and 200 μl DMSO was added, followed by 25 μl of Sorensen’s glycine buffer (0.1 M glycine, 0.1 M NaCl adjusted to pH 10.5 with 1 N NaOH). Subsequently, samples were then read in triplicate at 570/630 nm using an automated microplate reader (SpectraMax 340PC; Molecular Devices Corp.). The percent viability was calculated by the formula: (sample absorbance)/(control absorbance) x 100%.

Mitochondrial Membrane Potential Assay

ATI-like cells were plated and cultured on Lab-Tek chamber slides and treated with PBS (negative control), 3 μM valinomycin (Trevigen, Gaithersburg, MD) as a positive control, or 5% CSE for 24 h. DePsipher^TM^ Kit (Trevigen, Gaithersburg, MD) was used for the detection of mitochondrial membrane potential according to the manufacturer’s recommendations. This kit uses a cationic dye 5,5',6,6'-tetrachloro-1,1',3,3'-tetraethylbenzimidazolylcarbocyanine iodide to indicate the loss of mitochondrial potential. The dye enters cells and fluoresces bright red in its multimeric form within healthy mitochondria. When mitochondrial potential collapses in apoptotic cells, the DePsipher^TM^ reagent cannot accumulate within mitochondria and remains in the cytoplasm as a green fluorescent monomeric form. Briefly, 5 μg/ml DePsipher^TM^ solution was used in 1x reaction buffer with stabilizer solution. Media were removed from the cells, replaced with this mixture, and then incubated at 37^o^C for 15 min. Subsequently, cells were washed twice with prewarmed 1x reaction buffer with stabilizer solution and observed immediately under the fluorescent microscope using a long-pass filter (fluorescein and rhodamine).

**References**

1. Kosmider B, Loader JE, Murphy RC, Mason RJ (2010) Apoptosis induced by ozone and oxysterols in human alveolar epithelial cells. Free Radical Biology & Medicine 48: 1513-1524.
